# X-ray generation by fs-laser processing of biological material

**DOI:** 10.1364/BOE.499170

**Published:** 2023-10-09

**Authors:** P. Mosel, J. Düsing, S. Johannesmeier, M. Patzlaff-Günther, S. Fröhlich, J. Mapa, S. Kalies, J. Bahlmann, T. Püster, J. Vahlbruch, G. Dittmar, H. Merdji, M. Fajardo, A. Trabattoni, A. Heisterkamp, U. Morgner, M. Kovacev

**Affiliations:** 1 Leibniz University Hannover, Welfengarten 1, Hannover 30167, Germany; 2 Laser Zentrum Hannover e.V., Hannover 30419, Germany; 3 Lower Saxony Centre for Biomedical Engineering, Implant Research and Development (NIFE), 30625 Hannover, Germany; 4 Institute of Radioecology and Radiation Protection, Leibniz University Hannover, Hannover 30419, Germany; 5 Ingenieur-Büro Prof. Dr.-Ing. G. Dittmar, Aalen 73433, Germany; 6 LOA, ENSTA ParisTech, CNRS, Ecole Polytechnique, Université Paris-Saclay 828 Boulevard des Maréchaux, 91120, Palaiseau, France; 7 GoLP, Instituto de Plasmas e Fusão Nuclear, Instituto Superior Técnico, Universidade de Lisboa, 1049-001 Lisboa, Portugal; 8 Center for Free-Electron Laser Science CFEL, Deutsches Elektronen-Synchrotron DESY, Notkestr. 85, 22607 Hamburg, Germany

## Abstract

The use of ultrashort pulse lasers in medical treatments is increasing and is already an essential tool, particularly in the treatment of eyes, bones and skin. One of the main advantages of laser treatment is that it is fast and minimally invasive. Due to the interaction of ultrashort laser pulses with matter, X-rays can be generated during the laser ablation process. This is important not only for the safety of the patient, but also for the practitioner to ensure that the legally permissible dose is not exceeded. Although our results do not raise safety concerns for existing clinical applications, they might impact future developments at higher peak powers. In order to provide guidance to laser users in the medical field, this paper examines the X-ray emission spectra and dose of several biological materials and describes their dependence on the laser pulse energy.

## 1. Introduction

Medical laser applications have transformed modern medicine thanks to advances in the development of ultrashort pulse laser systems [1–4]. These laser systems deliver extremely short pulses of energy, in the pico- to femtosecond range, enabling many complex medical procedures to be performed with unprecedented precision, processing speed and minimal damage to surrounding tissue [5]. Other advantages over conventional tools, especially in the medical field, are that the laser itself has no tool abrasion and produces minimal amounts of waste. This results in faster recovery and less pain for the patient, making the laser an ideal tool for the medical field [6,7].

In medicine, lasers are often used in ophthalmology (e.g. Excimer laser with ns to ps pulse duration at 193 nm and femtosecond laser systems at 1053 nm) [8,9], dermatology (e.g. continuous and pulsed systems like CO_2_ laser with 10600 nm and Nd:YAG laser with 1064 nm or 532 nm) [10] and dentistry (e.g. femtosecond laser at 1064 nm or diode laser with 810 nm) [11,12] to cut, vaporize and treat tissue. A distinction is made between different intensity ranges. Low level-laser therapy (LLLT) is often used for pain relief, wound healing and acupuncture with intensities of about 10^−2^ to 10^0^ W/cm^2^ [13,14]. For dermatological treatments, such as hair removal and treatment of skin diseases, applied lasers are in the medium intensity range of 10^4^ to 10^8^ W/cm^2^ [15–17]. For surgical procedures such as eye surgery and tissue cutting, intensities around 10^13^ W/cm^2^ [18,19] are used.

One significant application of ultrashort pulse lasers in ophthalmology is LASIK surgery, where femtosecond lasers precisely reshape the cornea and correct vision problems [20,21]. They can also remove cataracts and treat certain retinal diseases [22]. Another medical application of pulsed lasers is in dermatology. These lasers can penetrate deep into the skin without damaging the surface, making them an ideal tool for skin resurfacing and rejuvenation, as well as for cosmetic and pharmaceutical research. In tattoo removal, picosecond lasers are used to target and fragment tattoo pigment [23,24].

Due to the nonlinear interaction with the tissue and a high tuneability, femtosecond lasers have also found widespread use in biomedical and bioengineering applications. They can be operated above or fine-tuned below the optical breakdown threshold. This can be applied for single cell surgery or even manipulation of subcellular structures, as in detail reviewed by Vogel et al. [25]. Another application of ultrashort pulse lasers is plasma-mediated ablation to cut large areas of bone very precisely [7,26].

There are many factors to be taken into account to ensure proper safety measures when using lasers in medicine. In addition to the laser parameters (wavelength, pulse duration and pulse energy), the generation of X-rays due to the laser interaction with the tissue through ablation must also be considered [27]. Generally, X-ray emission can occur during the ablation of material due to the formation of a plasma in the ablation process. There are two distinct ways of generating X-rays, both of which are driven by the high electron velocities occurring within the plasma. The first, Bremsstrahlung, is based on the diversion of these fast electrons in the ion potential of the plasma. This change in acceleration leads to the emission of electromagnetic radiation following a thermal distribution. In case of the plasma the peak of this distribution is in the X-ray regime due to the high kinetic energies of the electrons. The second mechanism is line emission. Here high-energy electrons remove an inner shell electron from an atom, triggering the transition of electrons from higher energy levels into the inner shell. This is visible as characteristic lines in the X-ray spectrum.

As medical technologies continue to develop and improve, commercial laser systems with high peak powers become readily available for medical applications. This has the potential to transform the way we diagnose and treat a wide range of medical conditions. Even with low pulse energies, ultrashort pulse laser systems achieve high intensities well exceeding 10^13^ W/cm^2^. In general in this intensity regime X-ray generation can occur in a variety of materials [28]. While X-ray emission during laser processing of metallic workpieces has already been investigated in several studies [29–31], a recent study investigated biological material where increased X-ray doses have been measured e.g. during the processing of teeth [32]. To assess the potential risk and integrate it into safety guidelines, not only the dose but also the radiation spectrum is a key factor, as absorption strongly depends on it.

The focus of this study is on low-picosecond and femtosecond pulsed laser systems as high intensities can lead to X-ray generation [33,34]. In this work, the X-ray dose and emitted spectrum are investigated at different laser intensities for a variety of biological targets. These include bone, skin, eyes and synthetic substitutes such as hydroxyapatite. It is shown that high dose rates can be produced by ultrashort pulse laser processing, even on biological material. In order to minimise potential risks to patients, it is important to provide guidance to healthcare professionals using laser systems on safe practices.

## 2. Experiments

**Fig. 1.**
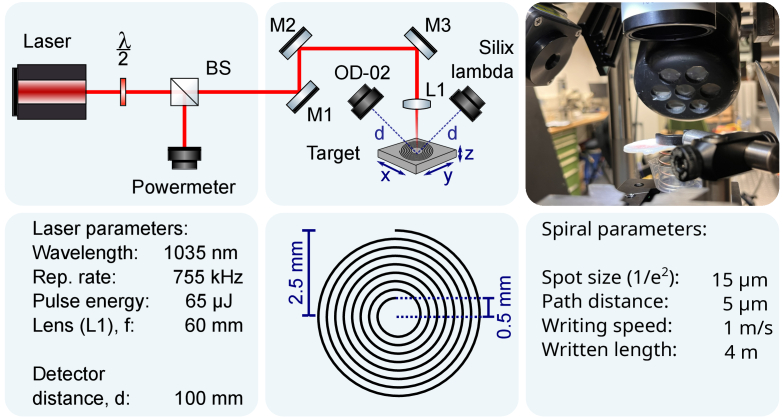
Schematic and a picture of the setup are shown at the top. Laser parameters and written pattern are shown bottom. The laser intensity can be adjusted using a waveplate. The emitted radiation is measured with the Silix Lambda and the OD-02 at a distance of 10 cm from the target.

The laser system used in this study is a commercial laser system (Monaco, Coherent) which has an adjustable repetition rate of up to 50 MHz. Here, a repetition rate of 755 kHz and a maximum pulse energy of 65 µJ with a pulse duration of 350 fs (1/e^2^) was used. During the experiments, the pulse energy was adjusted using an attenuator via a half-wave plate (Fig. 1). To achieve the required intensity in the material, the laser was focused with an f = 60 mm lens to a spot size of 15 µm. To remove possible debris, a suction device was placed behind the target. Depending on the sample material, one process cycle consists of 10 spirals with a radius of 2.5 mm or 5 mm were written on top of each other at a writing speed of 1 m/s and a path distance of 5 µm or 32 µm. This results in a total length of about 4 m or 2.46 m per spiral and ensures a gradual ablation of the target material without predominant scanning direction to average out directional dependent effects and formation of deep grooves.

During the experiments, an OD-02 dosimeter and a Silix lambda spectrodosimeter were used to simultaneously measure the X-ray dose and spectrum. The Silix has a detection range from 1.77 keV to 20 keV. Consequently, X-ray emission lines below 1.77 keV cannot be detected. This especially concerns the K_α_ lines of carbon, oxygen and nitrogen. Both detectors are aligned at an angle of 45° and placed at a distance of 10 cm from the sample (see Fig. 1). Since the expected radiation levels are relatively low, the total radiation dose in µSv is integrated during a process cycle of 10 spirals. The exact X-ray dose rate in µSv/h can then be calculated, with the total time of ablation for one such cycle. The dose and dose rate are given in H'(0.07) throughout the document, as the emitted radiation is in the low keV range. H’(0.07) describes the directional dose equivalent in the direction of maximum emission as defined by the International Commission on Radiological Protection (ICRP) 2007 recommendations after a in a depth of 0.07 mm in water. The background radiation integrated over one hour is measured as 0.2 µSv, which corresponds to a dose rate of 5 · 10^−5^ µSv/s. To be more comparable with the machining processes in medicine, the focus of the laser beam was placed 250 µm into the material.

Following the different applications of lasers, several biological samples were studied. All samples were obtained from pigs that were already sacrificed for other studies whereby the different samples were bone slices, skin and eyes (see Table 1). To prevent deterioration before the experiment, a phosphate buffered saline (PBS) solution was used to transport the samples and they were processed within a few hours after collection. Additionally, hydroxyapatite as an inorganic mineral present in human bone and teeth, and gelatin as a product of collagen extracted from skin, were tested as substitutes for real biological tissues. They are easily obtainable, chemically close to bones and skin respectively and could severely simplify future research by enabling preliminary experiments without the need for actual biological samples.

**Table 1.** Investigated biological materials and their chemical composition [39–41]

Material	Chemical composition	Water
Gelatin	C_X_H_Y_NO_Z_	15%
Skin (pig)	C_X_H_Y_NO_Z_ + Minerals	70%
Bones (pig)	CaO, MgO, P_2_O_5_, Ca/P	10%
Hydroxyapatite Alginat	Ca_10_(PO_4_)_6_(OH)_2_ (C_6_H_8_O_6_)_n_	0-99%
Eye	C_X_H_Y_N_Z_O_W_	70%

As a first step gelatin, skin and eye samples have been studied. The gelatin is from porcine skin (Sigma-Aldrich, G2625). Pig skin is similar in structure to human skin, but there are some differences in the composition of proteins and other molecules. For example, pig skin contains more collagen and less elastin than human skin. Overall, however, there are many similarities in the structure and function of pig and human skin, making it an often-used substitute for human skin in medicine [35]. Porcine corneas are used as a model for the eye as they are often used to replace human eyes in research and testing of eye treatments [36,37]. For the bone, several slices of 2 mm thickness were cut to achieve a flat top surface. This is essential as intensity fluctuations due to an uneven surface could affect the highly nonlinear process of X-ray generation. For measurements, multiple spiral patterns were written on the cortical bone from the top as well as the side to ensure consistency. The difference between human and pig bones is mainly that pig bones have a higher density and are therefore harder. In addition, it should be taken into account that pig bones are less porous and have a thicker cortex [38].

## 3. Results

### 3.1 Gelatin and skin

Gelatin is a protein complex produced from collagen by partial hydrolysis. As collagen is one of the main components of skin the chemical composition is close, however it does not contain e.g. some of the minerals present in skin. During the measurements, the gelatin samples were irradiated with 65µJ, the maximal energy in these experiments, but no increase in X-ray dose could be measured. Consequently, no emission spectrum could be acquired. There are two possible reasons for this. The first is the composition of gelatin itself, which only has K_α_ emission lines below the lower detection limit of the spectrometer (e.g. carbon at 277 eV or oxygen at 525 eV). Therefore, no line emission is expected from the sample. The second aspect is the absorption of air, which has an attenuation of > 10^6^ for photon energies of < 1.5 keV at a distance of 10 cm [42]. This means all potential signal from the line emission is absorbed before reaching the detector. This leaves only Bremsstrahlung, a type of radiation produced when charged particles are significantly slowed down or decelerated, which is not the case for gelatin.

**Fig. 2.**
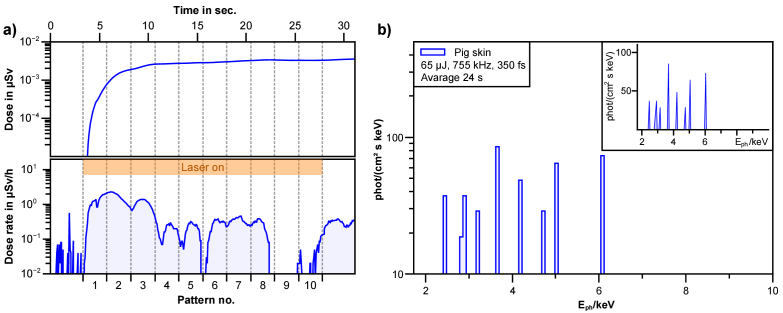
a) During laser treatment of porcine skin, the dose (top) and dose rate (bottom) were measured with the OD-02 over the treatment time and the written pattern. The orange bar indicates the process cycle with active laser irradiation (65 µJ). b) Shows the X-ray spectrum of a skin sample. The processing time was approx. 24 seconds with a pulse energy of 65 µJ. A low photon number at <100 photons / (keV cm^2^ s) was observed with the Silix lambda and reaches a dose rate of up to 2 µSv/h. The inset shows the linearly scaled spectrum.

In contrast to the “synthetic skin” made of gelatin, pig skin additionally contains a variety of minerals, e.g. sodium (Na, K_α_ = 1.04 keV), magnesium (Mg, K_α_ = 1.25 keV), potassium (K, K_α_ = 3.31 keV) or calcium (Ca, K_α_ = 3.69 keV). Since the potassium and calcium lines fall within the range of our detection devices, line emissions can be expected here. However, the flux should be extremely low due to the small concentration of minerals in skin. Therefore, the measured spectrum should be of little significance. This proved to be the case, as can be seen in Fig. 2(a). The X-ray dose is measured with the OD-02 over a series of spiral patterns with 2.5 mm diameter, written over a time period of about 24 seconds with a pulse energy of 65 µJ. The number of generated X-ray photons is low with < 100 photons / (keV cm^2^ s), with a peak dose rate of up to 2 µSv/h and an average dose rate of about 0.5 µSv/h over the processing time. When the laser is switched on, an immediate increase in the X-ray dose can be observed. Furthermore the single spirals can be seen in the dose rate (Fig. 2(a)). Consequently, only a few counts were measured for the spectrum in Fig. 2(b), which makes identification of the specific emission lines challenging. It is to be noted here, however that the measured spectrum and dose rate using the Silix is recorded close to the lower detection threshold and might therefore be indistinguishable from noise. Longer integration times could not be achieved due to the decomposition of the target material by laser irradiation.

### 3.2 Eyes

As laser treatments of the eye are already a common procedure, a study of potential hazards is especially critical. The focus here is on the cornea, as this is the area of the eye that is affected by laser treatment. It is mainly composed of collagen, wherefore, the emission spectrum should as well be similar to that of skin, but with a higher dose due to the firmer structure. Based on the results for skin, a low X-ray dose is expected. Due to this, a high pulse energy at 65 µJ is chosen, to enforce the generation of X-rays. It has to be noted though that the intensity is around six times higher than the usual energies of commercial lasers used for eye treatment [7,43]. Nevertheless, this marks an extreme case that can be set as an upper limit for the achievable X-ray doses and should show that it is generally possible to generate X-rays from the interaction of high intensity lasers and the cornea. For a lower laser intensity, as currently used in eye treatments, the X-ray emission would be much lower. Since the resulting photon energies are low energy radiation, which is almost absorbed within 70 µm, and for better comparison, H'(0.07) will be used even though for the human eye a specific dose rate value of H'(3) is usually considered.

**Fig. 3.**
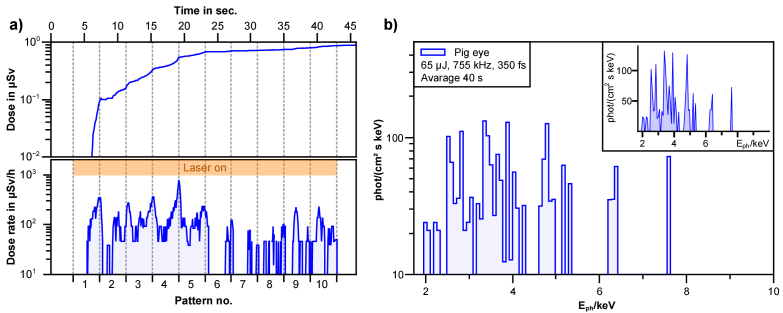
a) During laser treatment of a porcine eye, the dose (top) and dose rate (bottom) were measured with the OD-02 over the treatment time and the written pattern. The time when the laser (65 µJ) was on is marked in orange. b) Measured X-ray spectrum (blue) with the Silix lambda in logarithmic scale. In the corner the spectrum in linear scale.

The measurements were carried out as described before and the resulting spectra and doses are summarized in Fig. 3. After 20 second of continuous processing of the material a dose of 0.68 µSv is accumulated, resulting in an average dose rate of 122.4 µSv/h. This means a dose of 4.5 *·* 10^−14^ Sv per laser pulse. After this first interval, the dose rate decreases noticeable to about 37.6 µSv/h. This change is caused by material ablation, which shifts the material surface away from the focal spot of the laser. As in medical procedures, the focal spot is continuously adapted e.g. to cut material, only the first interval can be considered reliable. The measured X-ray spectrum with the characteristic lines for different elements is shown in Fig. 3(b). The emission spectrum does not show clear peaks, but some such as potassium (K, K_α_ = 3.31 keV) or calcium (Ca, K_α_ = 3.69 keV) are more likely to be expected.

### 3.3 Bones

The composition of bone is a combination of different materials, including minerals, collagen, proteins and water. While the exact chemical composition of bone can vary depending on the type and age of the bone, most of the bone mass is made up from minerals such as calcium phosphate (Ca_3_(PO_4_)_2_) and calcium carbonate (CaCO_3_) in the form of tiny crystals embedded in a collagen matrix. Additionally, water makes up about 15 - 25% of the bone mass. Based on this, expected characteristic lines from bones include especially those of phosphorus (P, K_α_ = 2.01 keV and K_β_ = 2.14 keV) and calcium (Ca, K_α_ = 3.69 keV and K_β_ = 4.01 keV).

For bone surface dosimetry the ICRP Publication 133 defines a target volume which is at a depth of 50 µm from the bone surface. Within the 50 µm layer there are bone precursor and bone stem cells [44]. In order to take the photon energy in the low keV range into account and to make comparable statements, units of H'(0.07) is used for the dose. Reversible metabolic bone diseases are reported at a radiation dose beginning of 1 Gy, but they are caused indirectly. For example Osteoradionecrosis occurs at doses higher than a few Gy. While inhibitions of bone growth are reversible and occur above 5 Gy, irreversible damage is expected above 10 Gy.

**Fig. 4.**
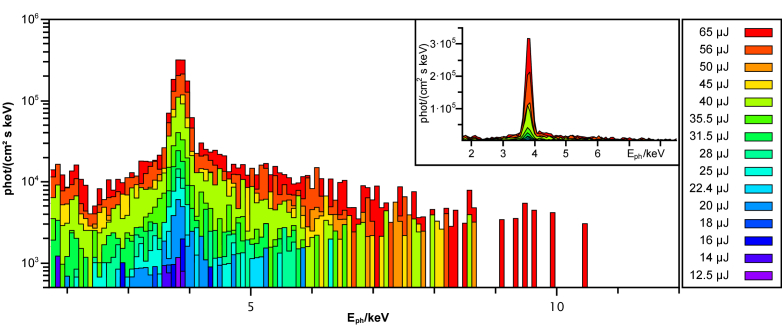
The X-ray spectrum of ablating bone is shown in logarithmic scale for different laser intensities between 12.5 µJ and 65 µJ. The characteristic lines through the elements phosphorus (P, K_α_ = 2.01 keV) and calcium (Ca, K_α_ = 3.69 keV) are visible. The inset shows the linearly scaled spectrum.

The laser power used for processing was varied between 12.5 µJ and 65 µJ to account for the increase in X-ray emission with intensity. The spectral measurements show a peak at 3.7 keV for all intensities (see Fig. 4). We attribute this to the K_α_ emission of calcium (Ca, K_α_ = 3.69 keV), which is consistent with its high concentration in bones. A second, much weaker peak can be seen at about 2.1 keV, which corresponds to the line emission of phosphorus. Additionally, a background extends from about 2.5 keV up to about 10 keV. The extend towards higher energies and dose of the background is increasing with the laser intensity.

**Fig. 5.**
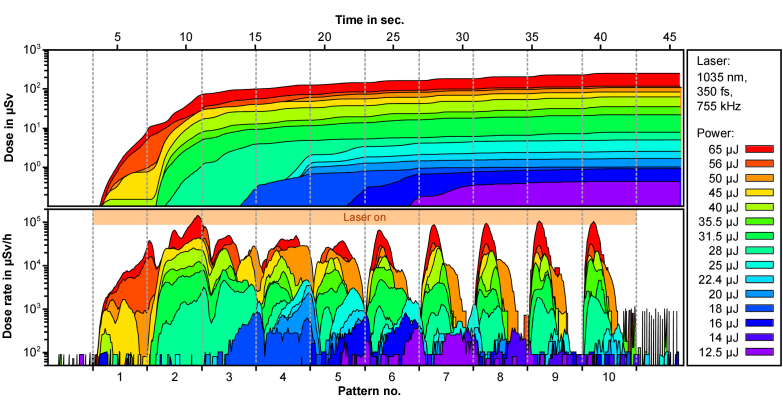
During laser treatment of a porcine bone, the dose (top) and dose rate (bottom) were measured with the OD-02 over the treatment time and the written pattern. The laser intensity was varied between 12.5 µJ and 65 µJ and the laser on times are marked in orange.

Besides the spectrum the emitted dose is a crucial parameter to estimate the potential danger from the emitted X-rays. Figure 5 shows the doses measured simultaneously with the OD-02 to the spectra. A clear increase of the dose is observed for each process cycle. During a writing process of 10 spirals, a dose (H'(0.07)) between 0.1 µSv (at 12.5 µJ) and 236 µSv (at 65 µJ) was measured within 40 seconds, depending on the laser intensity. This corresponds to a dose rate of 9 µSv/h to 21 mSv/h (H'(0.07)) or to a dose per pulse of approx. 3 *·* 10^−15^ Sv at 12.5 µJ up to 8 *·* 10^−12^ Sv at 65 µJ.

**Fig. 6.**
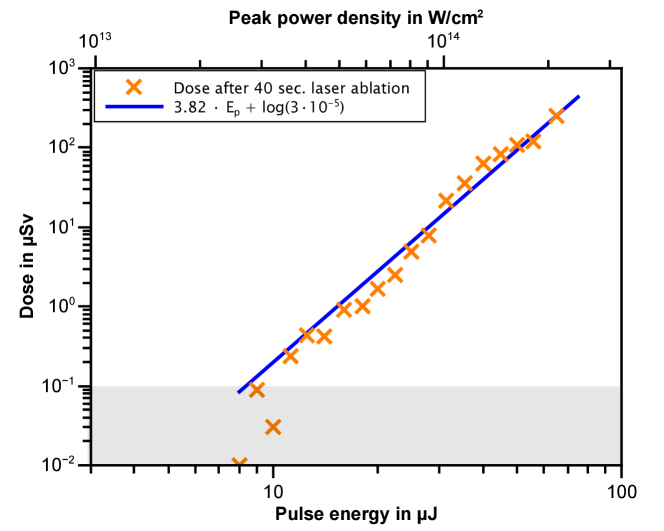
In orange, the dose of a 40 second laser treatment of bone is plotted for different intensities. A linear function is fitted in blue in the double logarithmic scale.

This clearly shows the strong intensity dependence of the X-ray dose. To condense this into an easier scaling law for quick estimates the intensity dependence of the total dose is depicted in Fig. 6. The relation closely follows a polynomial function, as the polynomial fit (blue line) illustrates. Thereby, the emitted dose is directly related to the pulse energy via *k · E_p_^a^*, where *k* depends on the material and laser parameters, while in our case the order of nonlinearity *a* corresponds to 3.82. This is a linear function in a double logarithmic graph with the fit 3.82 *· E_p_* + log(3 *·* 10^−5^). As long as the pulses can be considered as single pulses, the repetition rate of the laser goes linearly into the equation. Around a repetition rate of 1 MHz, pulse-to-pulse interaction has been observed in metals, which can have an effect on the emitted spectrum as well as an exponential effect on the dose. The 755 kHz repetition rate used here is close to pulse-to-pulse interaction, but the pulses can still be considered as single pulses [31,45].

### 3.4 Synthetic bone

**Fig. 7.**
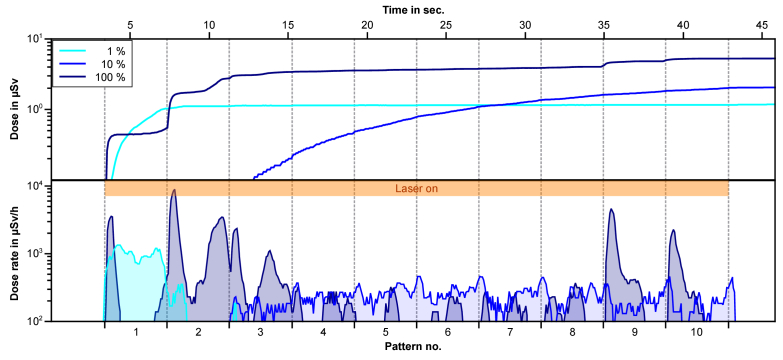
X-ray dose (top) and dose rate (bottom) over laser processing (65 µJ) time in seconds and written pattern number for different concentrations of hydroxyapatite in alginate. The 1% is 99% alginate with 1% hydroxyapatite powder – the 10% is 90% alginate with 10% hydroxyapatite powder – 100% is hydroxyapatite powder only. No X-rays were generated by laser ablation of pure alginate.

In medical applications, Ca_10_(PO_4_)_6_(OH)_2_ - based material are already used as biomaterials for bone replacement (bone grafting) or as a bioactive coating on implants to improve bone incorporation. Hydroxyapatite is the main component of bone and contributes significantly to the X-ray emission during laser processing as seen in Fig. 4, so the influence of the concentration of hydroxyapatite in the material and the X-ray emission produced is investigated. For different concentrations of hydroxyapatite in alginate, the X-ray emission as a function of processing time is shown in Fig. 7. To get different concentrations alginate (C_6_H_8_O_6_)_n_ was chosen as a carrier substrate for hydroxyapatite powder.

Laser processing, with 65 µJ, of a pure alginate sample, showed no measurable increase in X-ray dose. A concentration of 1% hydroxyapatite shows a peak in the dose rate only at the beginning of the laser ablation. This may be due to the powder forming a thin layer, which is completely ablated after a few cycles. Here, for the complete laser processing (65 µJ) a dose of 1.2 µSv and an average dose rate of about 105 µSv/h (H'(0.07)) is obtained. A higher concentration prevents the hydroxyapatite from settling. At a concentration of 10% and a processing time of 40 seconds, a dose of about 2 µSv (H'(0.07)) is produced. On average, this corresponds to a dose rate of 180 µSv/h (H'(0.07)). For powder the dose is 5.3 µSv after 40 seconds, resulting in an average dose rate of about 477 µSv/h.

**Fig. 8.**
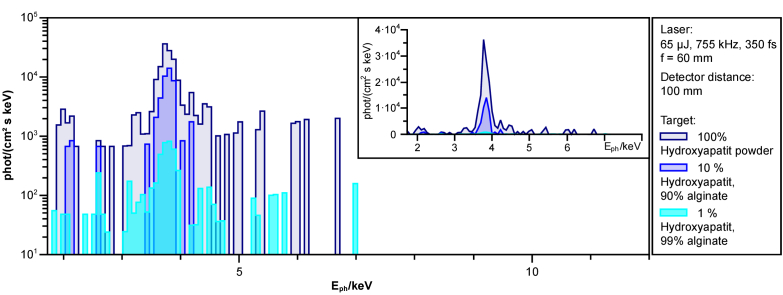
X-ray spectrum for different concentrations of hydroxyapatite in alginate. No X-rays were generated by laser ablation of pure alginate. Visible are the characteristic lines through the elements phosphorus (P, K_α_ = 2.01 keV) and calcium (Ca, K_α_ = 3.69 keV). The inset shows the linearly scaled spectrum.

Similar to bones, lines from phosphorus (P, K_α_ = 2.01 keV and K_β_ = 2.14 keV) and calcium (Ca, K_α_ = 3.69 keV and K_β_ = 4.01 keV) are expected in the emission spectrum of hydroxyapatite (Ca_5_[OH|(PO_4_)]_3_) during laser processing. Figure 8 shows the measured spectra for 1% and 10% hydroxyapatite in alginate and for the powder only, 100% hydroxyapatite. The support material in the form of alginate does not emit any measurable amount of X-ray radiation. In the emitted spectra, the K-alpha line of calcium is clearly visible. Meanwhile, the K_α_ line of phosphorus is fainter, but still clear.

## 4. Discussion

A multitude of laser and material parameters make it difficult to give an overall indication of the potential risks. However, it is important to make a distinction between the practitioner and the patient, as their distance to the point of interaction and exposure time is vastly different. To account for the gap in distance, an estimated dose rate at short distance from the laser interaction point is given for the patient and the dose rate for the hand and eye at 10 cm and 20 cm from the interaction point is given for the practitioner. Additionally, safety equipment plays an important role in the estimation of the dose. However, as this is a highly individual factor it requires extensive further studies and has not been accounted for in the present study. For a correct approximate dose, in addition to the 1/r^2^ reduction with distance, air absorption has to be taken into account. Consequently, the spectrum of the emitted X-rays becomes particularly important, as air has a strong absorption below 5 keV. This results in the patient being subject to high, localized doses of low energy X-ray radiation. The collected results of the measurements and the calculated values for different distances, taking into account 1/r^2^ and the absorption of air, are listed in Table 2. Note there are no specific limitation values for dose rates for patients.

**Table 2.** Investigated biological materials

Laser	1035 nm, 350 fs, 755 kHz			
Material	Pulse energy (µJ)	0.01 cm Calculated (mSv/s)	10 cm Measured (µSv/h)	20 cm Calculated (µSv/h)
Gelatin	65	-	**-**	-
Skin (pig)	65	1.78	**2**	0.16
Eye (pig)	65	109	**122.4**	9.5
Bones (pig)	8–65	8–18 940	**9–21 240**	0.7–1 654
Hydroxyapatite	65	425.3	**477**	37.2

### Gelatin and skin

Based on the measurements on skin and gelatin with laser parameters (65 µJ, 755 kHz) far above those normally used, a potential hazard to the practitioner from X-rays can be excluded. In combination with the measured spectrum, which is below 6 keV, the X-ray radiation can be limited to H’(0.07). An accumulated annual exposure time of 2000 hours results in a local dose of 4 mSv to the practitioners hand in 10 cm working distance. Here the estimate of 2000 hours exposure per year serves as an upper boundary that is likely above real exposure times. However, the dose to the patient remains an open question, as the radiation is largely absorbed by the surrounding tissue. This might result in a much higher local dose. It should be noted that the time during which the patient is exposed to X-rays is relatively short.

The following assumptions are made to estimate the dose rate close to the point of interaction: A distance of 0.01 cm from the laser interaction point was chosen because the laser spot has a focal spot of about 15 µm and up to 50% of the radiation is absorbed by about 70 µm of water. Thus, at a distance of 0.01 cm from the laser interaction point, the dose rate would have to be about 10^6^ times higher than if it was measured at 10 cm. Assuming that the X-ray photons have an energy of 3.7 keV, the air absorbs about 68.8% of the radiation within 10 cm. Since the point of interaction is on the surface of the patient, there is no shielding. This gives a dose rate to the patient of 6.4 Sv/h (1.78 mSv/s) or 2.4 nSv/pulse. The processing time for one spiral was about 4 s, which corresponds to a very local dose of about 7.1 mSv. However, as no clear spectrum could be measured, the uncertainty is very large.

### Eye

Using the same assumptions as for the skin, the dose rate to the patient at a distance of 0.01 cm from the point of interaction is approximately 393 Sv/h (109 mSv/s) or 145 nSv/pulse. Although a treatment lasts only a few seconds, the dose is close to the legal limit (20 mSv per year for occupational exposure [46]) for comparatively high laser parameters. In the real application, the intensity used is about 3 *·* 10^13^ W/cm^2^, which results in a reduction of the dose rate by about 10^3^ times under the assumption that the intensity dependence of the X-ray dose is similar to that of bone (see Fig. 6). This means that the expected dose rate to the patient's eye during LASIK is approximately 109 µSv/s, which corresponds to a local dose of 1.2 mSv for a treatment duration of 11 seconds [47]. A similar case can be made for the practitioner. The dose rate of 122.4 µSv/h at a distance of 10 cm with 65 µJ laser energy reaches 244.8 mSv for the hand in 2000h. However, it should be noted that both the working time at the laser and the laser power are set very high and are the exception. Assuming that the practitioner observes the laser treatment at a distance of 20 cm, a local dose rate of 9.5 µSv/h or 19 mSv/year can be estimated. By relaxing the working time and laser energy, the dose can be significantly reduced. Nevertheless, there is a risk to the practitioner which can be easily reduced by simple protection or awareness of the possibility of X-ray generation. This is in contrast to the patient where the close proximity severely limits the possible protection options. Additionally, the laser system and application need to be considered in each individual case. With the intensity dependence shown in Fig. 6, scaling is possible if other laser parameters such as the pulse length are kept constant.

### Bones and hydroxyapatite

Strong X-ray radiation can be measured during the laser treatment of bone with dose rates comparable to those in laser metal processing [28,48]. For the operator, the annual limit for the hand (500 mSv per year for occupational exposure [49]) would be reached within 24 hours of working time, using 65 µJ depending on the laser intensity. The dose rate to the patient is much higher due to the shorter distance and the lack of shielding. As the radiation is absorbed by the surrounding bone and skin in the immediate vicinity (0.01 cm) of the laser interaction point, the local dose rate to the patient is 6.8 · 10^4^ Sv/h (18.9 Sv/s) or 25 µSv/pulse, taking into account the increase due to the shorter distance and the missing absorption by the air. Even with shielding by surrounding tissue and assuming that the dose rate is considered at a distance of 1 mm, the local dose rate values are still at 4.7 µSv/h. At the same time, this shows that all the radiation was deposited within 1 mm of the point of interaction, as the radiation is in the soft X-ray range. At lower laser intensities in the range of 10^13^ W/cm^2^, the deposited local dose in bone with the laser used here would be about 0.01 µSv/pulse, which accumulates within a few seconds.

From the X-ray spectrum, the elements phosphorus and calcium can be identified as the main contributions (Fig. 4). These elements are found in hydroxyapatite and are responsible for the hardness of bone [38]. However, hydroxyapatite is also found in higher concentrations in teeth where laser treatment is already common. In addition to intensity (Fig. 6), the hydroxyapatite content also affects the X-ray radiation produced (Fig. 8).

The extent to which the measured dose rates can be transferred to other laser treatments remains an open question. Our results provide an upper limit at which a potential risk of X-ray emission occurs, for the patient as well as for the practitioner. It should be noted that the German legislation only applies from a photon energy above 5 keV. However, the measured X-ray emission of mainly 3.7 keV is only partially absorbed by air (absorption: 68.8% after 10 cm of air) and that this shielding is not provided for the patient. On the other hand, the treatment time for the patient is in the range of a few seconds [43].

## 5. Conclusion

X-rays are generated during laser treatment of tissue, eye and bone. This must be taken into account, particularly in the case of ultrashort pulse laser processing, as there is no distance between the point of interaction and the surrounding material, e.g. lens in the eye, tooth in the mouth or bone for in vivo surgery. This is important not only for the patient, because the safety precautions, distance, time, shielding, do not apply any more, but also for the person who performs or monitors the treatment. It should be noted that the X-ray radiation produced is strongly dependent on the laser parameters. While this work shows high dose rates can be produced by ultrashort pulse laser processing, even on biological material, it studies extreme cases above the currently used intensities. Nevertheless, we believe, based on our observations, that further studies are needed to clarify the potential risks further, especially if intensities of 10^13^ W/cm^2^ are exceeded. The spectrum plays a decisive role here: the Ca lines are below 4 keV and are therefore attenuated by, for example, a few centimeters of air. Nevertheless, it is still harmful radiation. Even simple protection can ensure that X-ray radiation is significantly reduced and risks are avoided for the practitioner. For the patient, especially when using intensities above 10^13^ W/cm^2^, secondary X-ray emission hazards should be taken into consideration.

## Data Availability

Data underlying the results presented in this paper are not publicly available at this time but may be obtained from the authors upon reasonable request.
